# Antioral Squamous Cell Carcinoma Effects of Carvacrol via Inhibiting Inflammation, Proliferation, and Migration Related to Nrf2/Keap1 Pathway

**DOI:** 10.1155/2021/6616547

**Published:** 2021-06-10

**Authors:** Hui Liu, Xiaoliang Xu, Ran Wu, Lei Bi, Chunguang Zhang, Hui Chen, Yang Yang

**Affiliations:** ^1^Department of Stomatology, North China University of Science and Technology Affiliated Hospital, Tangshan, 063000 Hebei, China; ^2^Department of Stomatology, The Second Hospital of Tangshan, Tangshan, 063000 Hebei, China

## Abstract

**Objective:**

To observe the therapeutic effect of Carvacrol on oral squamous cell carcinoma (OSCC) and dissect underlying molecular mechanisms.

**Methods:**

Keap1/Nrf2, NALP3, Vimentin, and E-cadherin expression was detected in OSCC and normal oral mucosa (NOM) tissues using immunofluorescence or western blot. When treated with Carvacrol or tert-butylhydroquinone (TBHQ) that activates Nrf2, the expression of Keap1/Nrf2/HO-1, epithelial-mesenchymal transition- (EMT-) related proteins, and NALP3 was examined in OSCC cells. Nrf2 was silenced by treatment with sh-Nrf2 or ML385. After silencing Nrf2 or Carvacrol treatment, cell proliferation and migration were assessed by clone formation and scratch and transwell tests in OSCC cells. Moreover, the expression of Keap1/Nrf2/HO-1, EMT-related proteins, and NALP3 was detected.

**Results:**

Keap1/Nrf2, NALP3, Vimentin, and E-cadherin proteins were all significantly upregulated in OSCC than NOM tissues. Carvacrol significantly suppressed Keap1/Nrf2/HO-1 activation. Carvacrol or silencing Nrf2 markedly inhibited the expression of Keap1/Nrf2/HO-1, EMT-related proteins, and NALP3 inflammasome in OSCC cells. Furthermore, clone formation and migration capacities were suppressed following treatment with Carvacrol or Nrf2 depletion. The opposite results were found when there is overexpression of Nrf2. However, Carvacrol distinctly improved the cancer-promoting effect induced by Nrf2 overexpression.

**Conclusion:**

Our findings suggested that Carvacrol ameliorated inflammation, proliferation, and migration for OSCC, which was related to inhibition of the Nrf2/Keap1 pathway.

## 1. Introduction

Oral squamous cell carcinoma (OSCC) is a heterogeneous cancer caused by the lining of the oral mucosa, which occupies 90% of all oral cancer cases globally [1]. Even though surgery combined with radiotherapy or chemotherapy has made great progress, OSCC patients exhibit the 5-year survival rate of <60% on account of local invasion, metastases, and recurrence [2]. Cetuximab, which targets the epidermal growth factor receptor, has been approved for treating OSCC [3]. It is still the only approved molecular targeted therapy for OSCC. However, high drug resistance limits the clinical application of Cetuximab. Immunotherapy, a promising strategy, has not yet achieved significant results in treating OSCC [4]. Hence, it is of importance to develop novel improved therapeutic strategies for OSCC.

Extensive studies have elucidated that continuous oxidative stress may induce chronic inflammation, thereby mediating most chronic diseases, especially cancers [5]. Nuclear factor erythroid 2-related factor (Nrf2) is a member of the CNC-bZIP transcription factor family [6]. Under normal physiological conditions, Nrf2 is combined with the cytoplasmic Kelch-like ECH-associated protein 1 (Keap1) that has ubiquitin ligase E3 activity [7]. Under the mediation of Keap1, Nrf2 is degraded by ubiquitination. When exposed to oxidative stress, the ubiquitin ligase E3 of Keap1 is inactivated, leading to the inactivation of the ubiquitination degradation of Nrf2 [8]. Subsequently, Nrf2 is transferred into the nucleus, thereby participating in the transcription of target genes. It has been accepted that Nrf2 plays a dual role in tumor progression. Recently, it has been reported that Nrf2 is upregulated in OSCC than normal tissues [9]. Its high expression can promote cancer phenotypes for OSCC cells [9]. The Keap1-Nrf2 pathway may mediate the expression of heme oxygenase-1 (HO-1) that plays protective functions in cells [10]. Upregulated HO-1 is in association with malignant progression of head and neck squamous cell cancer [11]. Hence, targeting the Keap1/Nrf2/HO-1 system could become a potential therapy strategy for OSCC.

Carvacrol is a natural-bioactive monoterpenoid phenol. It exhibits various pharmacological features against inflammation [12], oxidation [13], cancers [14], and so on. For example, Carvacrol exerts antioxidant and anti-inflammatory functions in colon cancer cells [15]. It ameliorates malignant biological behaviors of prostate cancer cells via targeting PI3K/Akt as well as MAPK pathways [16]. Nevertheless, its pharmacological effects on various hallmarks of OSCC cells remain undiscovered. Therefore, this study demonstrated that Carvacrol treatment markedly ameliorated inflammation, proliferation, and migration for OSCC cells, which was related to inhibition of the Nrf2/Keap1 pathway.

## 2. Materials and Methods

### 2.1. Patients and Specimens

61 pairs of OSCC and the matched NOM paraffin-embedded tissues were collected from the Department of Pathology, Affiliated Hospital of North China University of Technology, between 2015 and 2017. Clinicopathological characteristics of each patient (differentiation and lymph node metastasis) were also obtained. Each participant signed written informed consent. This research followed the guidelines of the Declaration of Helsinki and was approved by the Ethics Committee of North China University of Science and Technology Affiliated Hospital (2015022).

### 2.2. Immunofluorescent Staining

Following deparaffinization and hydration, specimens were cut into 4 *μ*m thickness. The sections were incubated with fluorescent substance-labeled primary antibodies against Keap1 (1 : 200; Proteintech; China; 60027-1-Ig), Nrf2 (1 : 100; Proteintech; China; 16396-1-AP), and HO-1 (1 : 100; Proteintech; China; 27282-1-AP) overnight at 4°C. Then, sections were incubated with secondary antibodies including Alexa Fluor® 488 Conjugate (1 : 100; #ZF-0512, ZSGB-BIO, China) and Alexa Fluor® 594 Conjugate (1 : 100, #ZF-0513, ZSGB-BIO, China) at room temperature for 2 h. The images were observed under a confocal microscope (Olympus, Japan).

### 2.3. Western Blot

Tissues or cells were lysed by RIPA lysis (Beyotime, Beijing, China) plus protease inhibitor and phosphatase inhibitor on ice for 20 min. Then, samples were centrifuged at 14000 rpm for 10 min at 4°C. The supernatant was collected, and protein concentration was determined using BCA reagent (Beyotime). Protein samples were separated by SDS-PAGE and transferred onto PVDF membrane. The membrane was blocked by 5% milk for 1 h and incubated with primary antibodies against Keap1 (1 : 1500; Proteintech; China; 60027-1-Ig), Nrf2 (1 : 1000; Proteintech; China; 16396-1-AP), HO-1 (1 : 1000; Proteintech; China; 27282-1-AP), Vimentin (1 : 1000; Proteintech; China; 10366-1-AP), N-cadherin (1 : 100; Abcam; USA; ab98952), E-cadherin (1 : 100; Abcam; USA; ab40772), Akt1 (1 : 1000; Proteintech; China; 10176-2-AP), Akt1 phospho S473 (1 : 1000; Abcam; USA; ab81283), mTOR (1 : 2000; Abcam; USA; ab32028), mTOR phospho S2448 (1 : 800; Abcam; USA; ab109268), NALP3 (1 : 1000; Proteintech; China; 19771-1-AP), and *β*-actin (1 : 2000; Proteintech; China; 20536-1-AP) at 4°C overnight. Afterwards, the membrane was incubated with HRP-labeled secondary antibodies (1 : 5000; ZSGB-BIO; China; ZB-2305, ZB-2301) at room temperature for 2 h. Target proteins were visualized via BeyoECL Plus (KGP1121; Nanjing KeyGen Biotech Co., Ltd., China).

### 2.4. Cell Culture

CAL-27 cells (ZQ0606; Shanghai Zhongqiao Xinzhou Biological Technology Co., Ltd., China; http://www.zqxzbio.com/Index/p_more/pid/2593.html) were cultured on the Minimum Essential Medium plus FBS, 1% P/S, and 1% GlutaMax under 5% CO_2_ and 37°C environment.

### 2.5. Transfection and Treatment

For silencing Nrf2 in CAL-27 cells, shRNAs targeting Nrf2 (Shanghai Genechem Co., Ltd., China) lentiviral plasmid were transfected into CAL-27 cells for 48 h. The transfection efficiency was verified by western blot. The sequences of sh-Nrf2 were as follows: shNrf2#1: GATCCCCGGCCCTGAAAGCACAGCAGAATTCAAGAGATTCTGCTGTGCTTTCAGGGTTTTTTG; shNrf2#2: GATCCCCGGCCTGCTACTTTAAGCCATTTTCAAGAGAAATGGCTTAAAGTAGCAGGTTTTTTG; shNC: GATCCCCGGTTCTCCGAACGTGTCACGTTTCAAGAGAACGUGACACGUUCGGAGAATTTTTTG. Furthermore, 5 *μ*M Nrf2 inhibitor ML385 (AbMole, USA) that was dissolved in PBS with 5% DMSO was used to inhibit the expression of Nrf2 in vitro. The concentration was selected partly based on whether it affected cell viability.

### 2.6. Cell Counting Kit-8 (CCK-8) Assay

CCK-8 assay was utilized to determine cell viability. CAL-27 cells were treated with Nrf2 activator TBHQ to overexpress Nrf2 [17]. CAL-27 cells treated with different concentrations of Carvacrol (0, 6.25, 12.5, 25, 50, and 100 *μ*M) and TBHQ (0. 5, 10, 20, 50, and 100 *μ*M) were seeded in 96-well plates (3 × 10^3^ cells/well). Each group had 6 replicate holes. The cells were cultured in a 37°C, 5% CO_2_ incubator. After 48 h, the original medium was discarded. The cells were treated with 100 *μ*l of medium containing 10% CCK-8 solution (Beyotime, Beijing, China) and cultured for 2 h. The absorbance value was detected at 450 nm on the microplate reader (Bio-Rad, California, USA).

### 2.7. Colony Formation

Transfected or treated cells were inoculated into a 6-well plate (3 × 10^3^ cells/well) and cultured for about 2 weeks. During this time, the medium was changed every 3 days. The cells were stained using Giemsa (500 *μ*l/well) for 15 min at room temperature. Three fields of view were randomly selected, and the number of colony formation was counted under a microscope (Olympus, Japan).

### 2.8. Wound Healing

After marking the back of the 6-well plate, each group of cells was inoculated into a 6-well plate (3 × 10^3^ cells/well). When the cells grew to about 80%, serum-free medium was replaced and cells were treated with 1 *μ*g/ml mitomycin C for 1 h. The cells of each group were marked and streaked with a 200 *μ*l micropipette tip. The surface cell debris was washed with serum-free medium, and serum-free medium was changed for culture. Cells were cultured in an incubator at 37°C and 5% CO_2_. After culturing for 72 h, images were photographed and observed under a microscope. ImageJ software was utilized to quantify the migrated distance of each group.

### 2.9. Transwell Assay

The treated or transfected cells were digested with 0.25% trypsin and made into a single cell suspension. Then, these cells were inoculated into the upper chamber at a concentration of 3 × 10^3^ cells/well. Meanwhile, the lower chamber was added with 10% serum-containing conditioned medium. After 48 h, the cells were fixed and stained with crystal violet. The number of migrated cells was counted under a microscope.

### 2.10. Cellular Immunofluorescence

CAL-27 cells were seeded in a 24-well plate (3 × 10^3^ cells/well). Following fixing cells for 15 min, the cell-climbing slide was washed using 0.5% Triton× 100 PBS solution. Then, sections were blocked with 3% BSA and incubated with primary antibodies against Keap1 (1 : 200; Proteintech; China; 60027-1-Ig), Nrf2 (1 : 100; Proteintech; China; 16396-1-AP), and HO-1 (1 : 100; Proteintech; China; 27282-1-AP) overnight at 4°C, followed by incubation with secondary antibodies including Alexa Fluor® 488 Conjugate (1 : 100; #ZF-0512, ZSGB-BIO, China) and Alexa Fluor® 594 Conjugate (1 : 100, #ZF-0513, ZSGB-BIO, China) at room temperature for 2 h. The images were investigated under a confocal microscope (Olympus, Japan).

### 2.11. Statistical Analysis

GraphPad Prism 7 software (GraphPad Prism, USA) was utilized for statistical analysis. Data are presented as mean ± standard deviation. By normality test, all data followed Gaussian distribution. Comparisons with different groups were analyzed using paired Student's *t*-test or one-way analyses of variance followed by Tukey's multiple comparison test. Differences with ^∗^*p* < 0.05, ^∗∗^*p* < 0.01, ^∗∗∗^*p* < 0.001, and ^∗∗∗∗^*p* < 0.0001 were considered significant.

## 3. Results

### 3.1. Oxidative Stress, Inflammation, and EMT in OSCC

**Figure 1:**
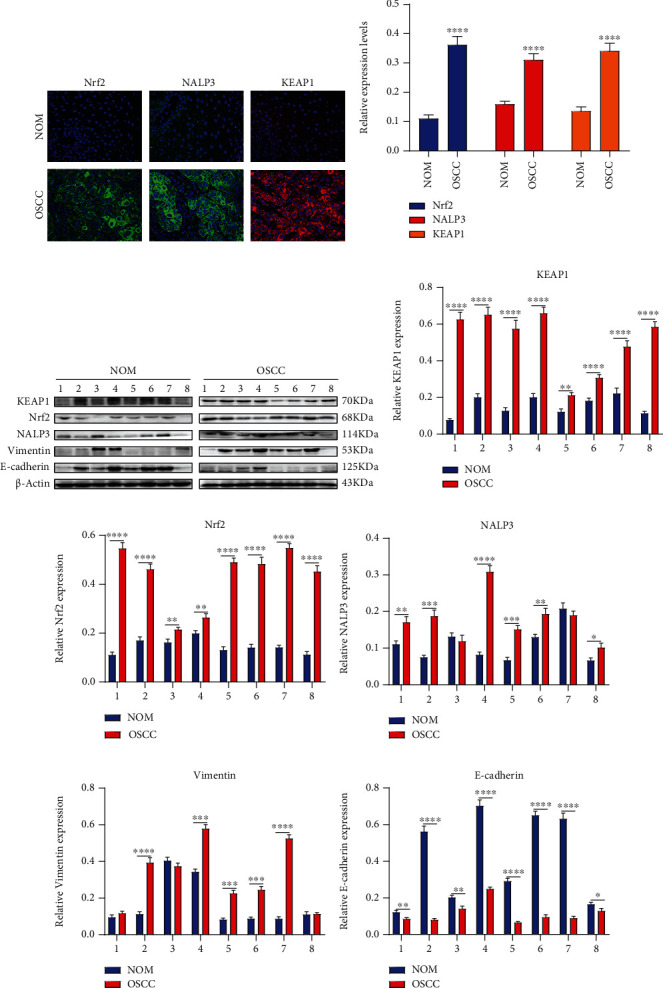
Abnormal expression of antioxidative stress-, inflammation-, and EMT-related proteins in OSCC tissues. (a, b) Immunofluorescence detecting the expression of Nrf2, NALP3, and KEAP1 between OSCC and NOM tissues. Bar = 20 *μ*M. (c) Western blot results examining the expression of (d) KEAP1, (e) Nrf2, (e) NALP3, (g) Vimentin, and (h) E-cadherin between OSCC and NOM tissues. For each experiment, *n* = 3. ^∗^*p* < 0.05; ^∗∗^*p* < 0.01; ^∗∗∗^*p* < 0.001; ^∗∗∗∗^*p* < 0.0001.

Aberrations in genetic expression patterns contribute to the pathogenesis of OSCC. Herein, we examined the expression of oxidative stress-, inflammation-, and EMT-related proteins in OSCC and NOM tissues. The immunofluorescence showed that Nrf2 and Keap1 expression was upregulated in OSCC tissues compared to NOM tissues (Figures 1(a) and 1(b)). Furthermore, NALP3 inflammasome also exhibited a higher expression level in OSCC than NOM tissues (Figures 1(a) and 1(b)). Western blot results confirmed the higher expression of Keap1 (Figures 1(c) and 1(d)), Nrf2 (Figures 1(c) and 1(e)), and NALP3 (Figures 1(c) and 1(f)) in OSCC specimens in comparison to NOM specimens. Moreover, the expression of EMT-related proteins including Vimentin and E-cadherin was determined between OSCC and NOM tissues via western blot. Higher Vimentin (Figure 1(g)) and lower E-cadherin (Figure 1(h)) expression was found in OSCC than NOM tissues. The above results reflected that antioxidative stress, inflammation, and EMT processes occurred during OSCC progression.

### 3.2. Clinical Features of Nrf2 and NALP3 in OSCC

The expression of Nrf2 and NALP3 was detected in 61 pairs of OSCC and the matched NOM specimens using immunohistochemistry. In Table 1, upregulation of Nrf2 and NALP3 was more frequently found in OSCC tissues than NOM tissues. The correlation between Nrf2 or NALP3 expression and clinicopathological characteristics was further analyzed. Consequently, Nrf2 or NALP3 expression exhibited a significant correlation with tumor differentiation and lymph node metastasis, indicating that Nrf2 and NALP3 might be underlying prognostic markers for OSCC.

### 3.3. Carvacrol Inhibits Keap1/Nrf2/HO-1 and EMT Pathways in OSCC Cells

**Figure 2:**
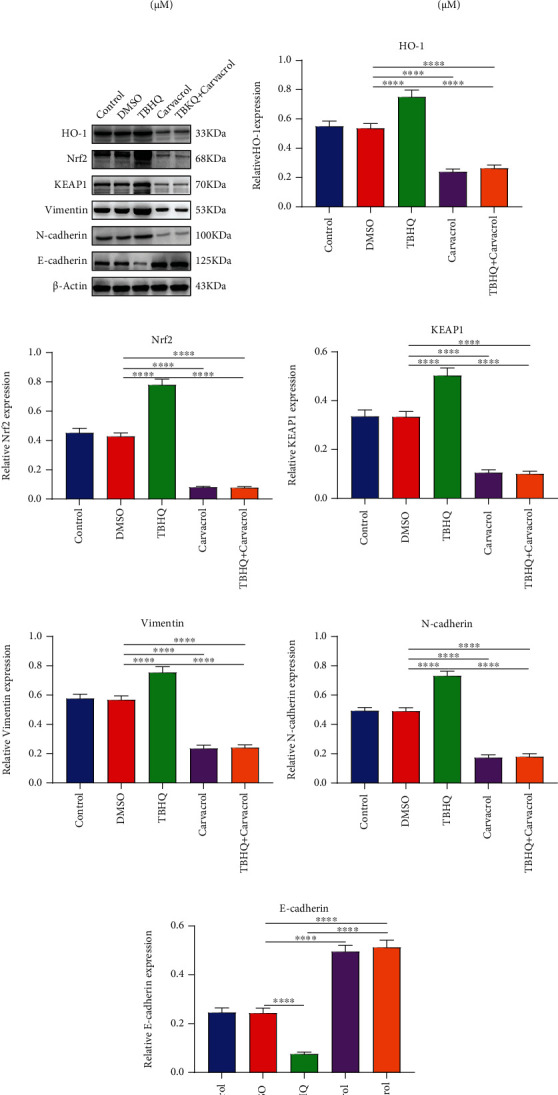
Carvacrol suppresses Keap1/Nrf2/HO-1 and EMT pathways in OSCC cells. (a, b) CCK-8 was utilized to determine the optimal concentration of Carvacrol and TBHQ in OSCC cells. (c) Western blot was presented to detect the expression of (d) HO-1, (e) Nrf2, (f) KEAP1, (g) Vimentin, (h) N-cadherin, and (i) E-cadherin in OSCC cells treated with Carvacrol and/or TBHQ. For each experiment, *n* = 3. ^∗∗∗∗^*p* < 0.0001.

To determine the optimal concentrations of Carvacrol and TBHQ (an antioxidant that activates Nrf2), we presented CCK-8 assay in CAL-27 cells treated with different concentrations of Carvacrol and TBHQ. 12.5 *μ*M and 20 *μ*M were selected as the optimal concentrations of Carvacrol (Figure 2(a)) and TBHQ (Figure 2(b)), respectively. We further observed the effect of Carvacrol on Keap1/Nrf2/HO-1 and EMT signaling pathways (Figure 2(c)). Our data suggested that TBHQ significantly increased the expression of HO-1 (Figure 2(d)), Nrf2 (Figure 2(e)), and Keap1 (Figure 2(f)) proteins in CAL-27 cells. Oppositely, Carvacrol distinctly decreased their expression in OSCC cells with or without TBHQ. Furthermore, the expression of Vimentin (Figure 2(g)), N-cadherin (Figure 2(h)), and E-cadherin (Figure 2(i)) was elevated in CAL-27 cells following treatment with TBHQ. The opposite results were investigated when treated with Carvacrol. Hence, Carvacrol could inactivate Keap1/Nrf2/HO-1 and EMT pathways in OSCC cells.

### 3.4. Carvacrol Suppresses AKT/mTOR Pathway and NALP3 Inflammasome in OSCC Cells

**Figure 3:**
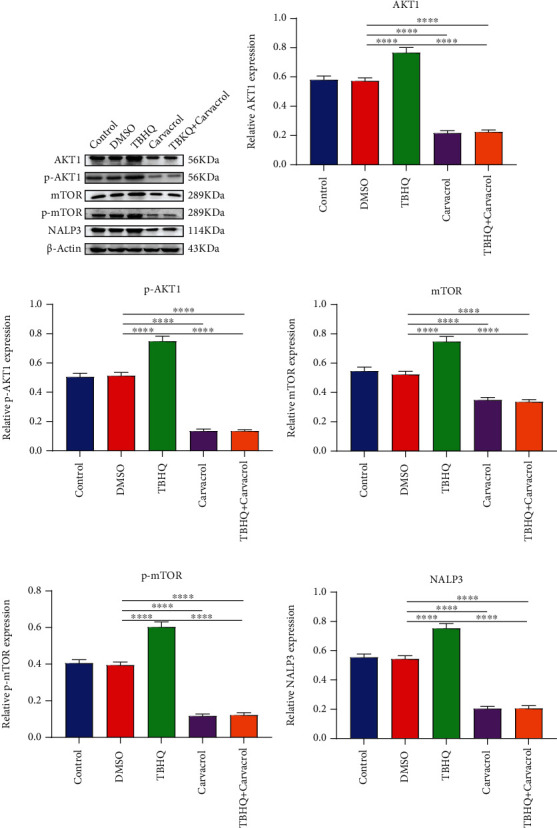
Carvacrol treatment inactivates the AKT/mTOR pathway and NALP3 inflammasome in OSCC cells. (a) Western blot was utilized to examine the expression of (b) AKT1, (c) p-AKT1, (d) mTOR, (e) p-mTOR, and (f) NALP3 in CAL-27 cells treated with TBHQ and/or Carvacrol. For each experiment, *n* = 3. ^∗∗∗∗^*p* < 0.0001.

We further investigated the effects of Carvacrol on the AKT/mTOR pathway and NALP3 inflammasome in OSCC. As shown in western blot results (Figure 3(a)), the expression of AKT1 (Figure 3(b)), p-AKT1 (Figure 3(c)), mTOR (Figure 3(d)), and p-mTOR (Figure 3(e)) was distinctly increased in CAL-27 cells treated with TBHQ. However, Carvacrol markedly inhibited their expression levels in OSCC cells with or without TBHQ. NALP3 expression was distinctly elevated by TBHQ treatment (Figure 3(f)). Carvacrol treatment ameliorated TBHQ-induced increase in NALP3 expression. Therefore, Carvacrol could inhibit the AKT/mTOR pathway and NALP3 inflammasome in OSCC cells partly via inhibition of Nrf2.

### 3.5. Carvacrol Suppresses Proliferation of OSCC Cells Partly by Nrf2

**Figure 4:**
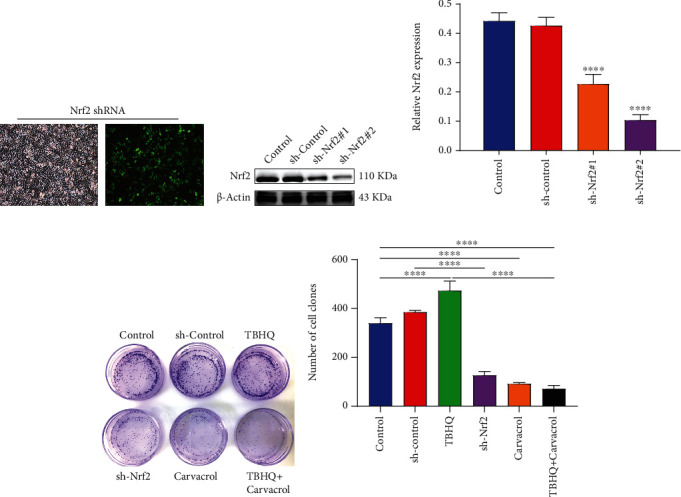
Carvacrol or inhibition of Nrf2 suppresses proliferation of OSCC cells partly by Nrf2. (a) Representative images of OSCC cells transfected with sh-Nrf2. (b, c) Transfection effects were assessed in OSCC cells transfected with sh-Nrf2. (d, e) Clone formation assay was utilized to evaluate the proliferative ability of OSCC cells transfected with sh-Nrf2 and treated with TBHQ and/or Carvacrol. For each experiment, *n* = 3. ^∗∗∗∗^*p* < 0.0001.

CAL-27 cells were transfected by sh-Nrf2 plasmid (Figure 4(a)). Western blot results confirmed that Nrf2 expression was successfully silenced in OSCC cells (Figures 4(b) and 4(c)). sh-Nrf2#2 was used for further experiments. Clone formation experiment results demonstrated that TBHQ treatment enhanced the capacity of clone formation for OSCC cells (Figures 4(d) and 4(e)). Nrf2 knockdown or Carvacrol treatment distinctly weakened the clone formation ability of OSCC cells. Moreover, Carvacrol improved TBHQ-induced enhancement in proliferation for OSCC cells. Collectively, Carvacrol suppressed the proliferative ability of OSCC cells, which was partly related to Nrf2.

### 3.6. Carvacrol Suppresses Migration of OSCC Cells Partly via Inhibition of Nrf2

**Figure 5:**
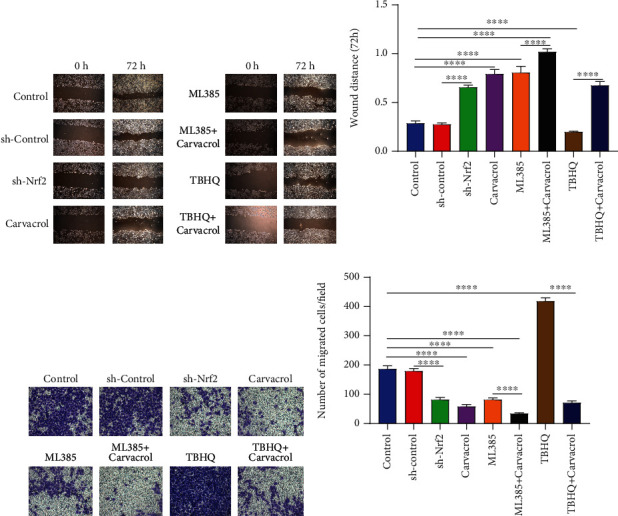
Carvacrol treatment suppresses migration of OSCC cells partly by Nrf2. (a, b) Wound distance was assessed in OSCC cells treated with sh-Nrf2, Carvacrol, ML385, or TBHQ. (c, d) Transwell assay was presented to examine the number of migrated cells when treated with sh-Nrf2, Carvacrol, ML385, or TBHQ. For each experiment, *n* = 3. ^∗∗∗∗^*p* < 0.0001.

The ability of cell migration was evaluated by scratch and transwell tests. After silencing Nrf2 by ML385 (a Keap1-Nrf2 inhibitor) or sh-Nrf2, the wound distance was distinctly longer compared to controls (Figures 5(a) and 5(b)). The similar results were found following treatment with Carvacrol. Under cotreatment with ML385 and Carvacrol, the wound distance was markedly longer than treatment with ML385. On the contrary, TBHQ treatment significantly shortened the wound distance, which was ameliorated by Carvacrol. Transwell assay results showed that Nrf2 knockdown and/or Carvacrol treatment significantly deceased the number of migrated cells (Figures 5(c) and 5(d)). Oppositely, the number of migrated cells was markedly increased when treated with TBHQ. As expected, Carvacrol ameliorated TBHQ-induced increase in the number of migrated cells. Taken together, Carvacrol could suppress the migrated ability of OSCC cells partly via Nrf2.

### 3.7. Carvacrol Inhibits NALP3 Inflammasome and EMT Process in OSCC Cells Partly through Nrf2

**Figure 6:**
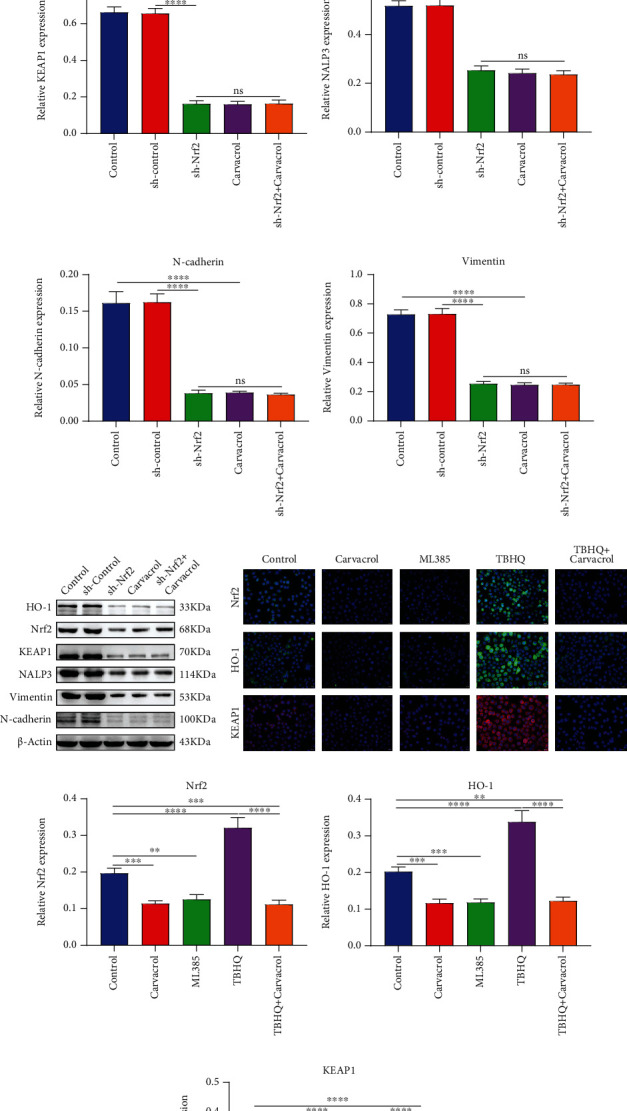
Carvacrol inhibits EMT process in OSCC cells partly by Nrf2. (a) HO-1, (b) Nrf2, (c) Keap1, (d) NALP3, (e) N-cadherin, and (f) Vimentin expression was detected in OCC cells treated with sh-Nrf2 and/or Carvacrol. (g) Representative images of western blots. (h) Immunofluorescence was utilized to detect the expression of (i) Nrf2, (j) HO-1, and (k) Keap1 in OSCC treated with ML385, Carvacrol, and/or TBHQ. For each experiment, *n* = 3. ns: not significant; ^∗∗^*p* < 0.01; ^∗∗∗^*p* < 0.001; and ^∗∗∗∗^*p* < 0.0001.

Silencing Nrf2 or Carvacrol markedly lowered the expression of HO-1 (Figure 6(a)), Nrf2 (Figure 6(b)), and Keap1 (Figure 6(c)) in OSCC cells, indicating that Carvacrol could inhibit the activation of the Keap1/Nrf2/HO-1 pathway. Furthermore, NALP3 expression was distinctly decreased following treatment with sh-Nrf2 or Carvacrol (Figure 6(d)). N-cadherin and Vimentin expression levels were also suppressed by sh-Nrf2 or Carvacrol treatment (Figures 6(e)–6(g)). These results suggested that Carvacrol could suppress NALP3 inflammasome and EMT process in OSCC cells partly by inhibition of Nrf2. We further examined Keap1, Nrf2, and HO-1 proteins in OSCC cells using immunofluorescence (Figure 6(h)). Our data demonstrated that Carvacrol or ML385 distinctly decreased the expression of Keap1 (Figure 6(i)), Nrf2 (Figure 6(j)), and HO-1 (Figure 6(k)) proteins in OSCC cells. TBHQ treatment distinctly elevated their expression levels in OSCC cells, which was ameliorated by Carvacrol (Figures 6(i)–6(k)). Therefore, Carvacrol treatment inhibited NALP3 inflammasome and EMT process in OSCC cells partly through Nrf2.

## 4. Discussion

In this study, our data suggested that Carvacrol inhibited the Keap1/Nrf2/HO-1 pathway in OSCC cells. Carvacrol treatment or suppression of Nrf2 both distinctly weakened proliferative and migrated capacities, NALP3 inflammasome, and EMT activation of OSCC cells. Carvacrol ameliorated proliferation, migration, inflammation, and EMT of OSCC cells after overexpression of Nrf2 induced by TBHQ. Thus, Carvacrol alleviated OSCC cell proliferation, inflammation, and migration partly by Nrf2.

Tumor cells are characterized by high proliferation and migration ability [18]. Previous work has reported the inhibitory effects of Carvacrol on proliferation and invasion of OSCC cells [19]. Consistently, our results demonstrated that Carvacrol repressed proliferation, migration, and the EMT pathway in OSCC cells. Previously, the function of Carvacrol on antitumor cell proliferation and migration has been observed in several cancers including non-small-cell lung cancer (NSCLC) [20], prostate cancer [21], and colon cancer [22]. Nrf2 is a critical transcriptional factor against oxidative stress or electrophiles. Consistent with previous work, our data showed that Nrf2-Keap1 system was activated in OSCC tissues [9]. Combining clinicopathological information, Nrf2 expression was positively correlated to differentiation and lymph node metastasis of OSCC, indicating that it could be an underlying prognostic factor. Recently, Nrf2 could become an independent predictor for clinical outcomes of hepatocellular carcinoma [23]. Overexpression of Nrf2 promoted OSCC cell proliferation and migration, and the opposite results were found when there was depletion of Nrf2. Intriguingly, Carvacrol treatment inactivated the Nrf2/Keap1/HO-1 pathway in OSCC cells. Herein, Carvacrol could ameliorate the effects of Nrf2 on promoting OSS progression, suggesting that Carvacrol exerts anti-OSCC roles partly via the Nrf2/Keap1 system.

Inflammation occurs in multiple stages of OSCC progression [24]. The NALP3 inflammasome can mediate inflammatory response within cancer tissues [25]. Our study firstly reported that NALP3, the key component of inflammasome [26], was upregulated in OSCC tissues by immunofluorescence as well as western blot. There were positive correlations between NALP3expression and tumor differentiation and lymph node metastasis among OSCC patients. A previous study has reported the correlation between its expression and prognosis, TNM stage, and differentiation for NSCLC [27]. High expression of NALP3 has been detected in NSCLC specimens, and its depletion could suppress NSCLC cell growth [27]. Previous work has reported the anti-inflammatory effects of Carvacrol on colon cancer cells [15]. Herein, we found that Carvacrol inhibited the expression of NALP3 protein in OSCC cells, indicating that Carvacrol could repress inflammation via blocking NALP3 inflammasome. The expression of NALP3 was elevated by Nrf2 overexpression induced by TBHQ and was decreased by Nrf2 depletion in OSCC cells. However, Carvacrol treatment could restrain inflammatory response due to Nrf2 overexpression.

The AKT/mTOR pathway is widely involved in OSCC progression [28]. Its activation mediates a variety of hallmarks of cancers, such as proliferation, migration, and metastasis [4]. Herein, Carvacrol treatment decreased AKT/mTOR pathway activation in OSCC cells. The opposite results were found in OSCC cells following overexpression of Nrf2, indicating that Carvacrol could inhibit OSCC cell proliferation and migration via the AKT/mTOR pathway. Loss of E-cadherin and increase in Vimentin and N-cadherin expression occur during EMT process [29]. EMT contributes to invasion and metastasis for OSCC cells [30]. Herein, we investigated the abnormal expression of EMT-related proteins in OSCC than NOM tissues. Both Carvacrol and Nrf2 knockdown markedly suppressed EMT activation for OSCC cells. Nrf2 overexpression accelerated EMT process. Previously, Nrf2 has been confirmed to promote EMT process for various cancer cells. For instance, Nrf2 activation of macrophages can induce EMT process in hepatocellular carcinoma cells [31]. Furthermore, it can facilitate breast cancer cell migration via the EMT pathway [32]. Hence, in OSCC cells, Carvacrol or Nrf2 depletion could induce cellular migration by activation of EMT process. Moreover, Carvacrol significantly reversed Nrf2 overexpression-induced increase in migrated capacity and EMT activation for OSCC cells, indicating that Carvacrol could repress OSCC cell migration and EMT by suppression of Nrf2.

Collectively, our results demonstrated that Carvacrol exerted anti-OSCC effects via inhibiting proliferation, inflammation, and migration, which was related to the Nrf2-Keap1 pathway.

## 5. Conclusion

In this study, we investigated the inhibitory effects of Carvacrol treatment on OSCC cells and further analyzed the underlying mechanism. Our data demonstrated that Carvacrol repressed proliferation, inflammation, and migration for OSCC cells partly via inhibiting the Nrf2/Keap1 system. In-depth studies require to be presented to confirm the therapeutic effects of Carvacrol on OSCC.

## Figures and Tables

**Table 1:** Clinical characteristics of Nrf2 and NALP3 expression for OSCC patients.

Characteristics	*n*	Nrf2			NALP3		
		+	−	Positive (%)	+	−	Positive (%)
NOM	61	39	22	63.9^∗^	33	28	54.1^∗^
OSCC	61	45	16	73.8	47	14	77.1
Differentiation							
Well	17	13	4	76.5%^∗^	15	2	88.2%^∗^
Moderately	24	18	6	75%	21	3	87.5%
Poorly	20	14	6	70%	15	5	75%
Lymph node metastasis							
Positive	25	18	7	72%^∗^	21	4	84%^∗^
Negative	36	26	10	72.2%	31	5	86.1

Abbreviations: OSCC: oral squamous cell carcinoma; NOM: normal oral mucosa. ^∗^*p* < 0.05.

## Data Availability

The datasets analyzed during the current study are available from the corresponding author on reasonable request.

## Abbreviations

OSCC: Oral squamous cell carcinoma
NOM: Normal oral mucosa
TBHQ: Tert-butylhydroquinone
EMT: Epithelial-mesenchymal transition
Nrf2: Nuclear factor erythroid 2-related factor
Keap1: Kelch-like ECH-associated protein 1
HO-1: Heme oxygenase-1
CCK-8: Cell Counting Kit-8.
